# Radiology of the Extemities

**Published:** 1992-03

**Authors:** Tom Snow


### RADIOLOGY OF THE EXTREMITIES

**Nancy Baker**

The tasteful turquoise and black design, the substantial last-a-lifetime hardcover, and the high gloss paper make this a coffee table textbook — friends will finger it with awe and wonder how much such a slim volume could have set you back $49.95. The text is less daunting than the packaging, and, unusually, Nancy Baker plunges into the subject on page 1 without any kind of preliminary fluff about imaging techniques or radiological philosophy. The illustrations are fine, especially the radiographs, and everything is set out very generously with expanses of blank page to flatter your reading rate — with a break to put the kettle on you could still be finished in two hours: not enough for radiology registrars but probably good for orthopaedic juniors and very keen medical students. This 1st edition has multiple typographical errors to mar the otherwise impressive presentation — pictures with the wrong captions, a diagram with no labels — and, tragically, the radiograph on the front cover is printed upside down! Overall, a book to admire and skim through, but best to borrow rather than buy.

Tom Snow

